# Special issue: Applications of probiotics

**DOI:** 10.3934/microbiol.2025040

**Published:** 2025-12-11

**Authors:** Einar Ringø, Srirengaraj Vijayaram, Yun-Zhang Sun

**Affiliations:** 1 Norwegian College of Fishery Science, Faculty of Bioscience, Fisheries, and Economics, UiT the Arctic University of Norway, Tromsø, 9037, Norway; 2 Xiamen Key Laboratory for Feed Quality Testing and Safety Evaluation, Fisheries College, Jimei University, Xiamen 361021, China; 3 Centre for Global Health Research, Saveetha Medical College and Hospital, Saveetha 7 Institute of Medical and Technical Sciences, Saveetha University, Thandalam, Chennai- 602 8 105, Tamil Nadu, India

Since the first paper was published using the word probiotics by Lilly and Stillwell [1] more than 54.000 papers have been published according to Web of Science. Probiotics are live organisms present in food [2],[3], human and animal gastrointestinal (GI) tract [4],[5]. The International Scientific Association of Probiotics and Prebiotics recommendations for the scope probiotics, see Table 1.

The probiotic microorganisms or friendly microbes, and they provide numerous health-beneficial effects to the host such as inhibition of adherence and colonization of pathogens, enhanced immune system, reduction of toxins, reduction of inflammatory bowel diseases, reduce cholesterol levels, prevention of cancer, synthesis of vitamins, and production of antimicrobial compounds [5]–[8]. Consumer awareness is the main reason for the development of probiotic functional food products. In this regard, probiotics incorporated in food products significantly enhanced the quality, taste, and flavour compared to non-probiotic functional foods. Furthermore, probiotic food products protect against unfavourable conditions and increased shelf-life periods [9],[10].

Probiotics with their consolidated history represent an excellent resource of natural products used as an alternative therapy, representing an essential component of traditional medicines. Therefore, the use of probiotics can be a promising alternative for the control of infectious diseases [11],[12]. Probiotics with conventional antimicrobial activity offers another field of application and can be widely pursued as a therapeutic approach capable of sensitizing resistant pathogens and contributing to limiting the antimicrobial resistance pandemics and the global burden of infectious diseases. Finally, probiotics are generally considered safe for animals, humans, and the environment [13].

**Table 1.** Consensus panel recommendations for the scope of probiotics. After Hill et al. [14].

No	Consensus panel recommendations
1	Retain the FAO/WHO definition for probiotics, with a minor grammatical correction as “live microorganisms that, when administered in adequate amounts, confer a health benefit on the host”; inconsistences between the Expert Consultation1 and the FAO/WHO Guidelines were clarified
2	Include in the framework for definition of probiotics microbial species that have been shown in properly controlled studies to confer benefits to health
3	Any specific claim beyond “contains probiotics” must be further substantiated
4	Keep live cultures, traditionally associated with fermented foods and for which there is no evidence of a health benefit, outside the probiotic framework
5	Keep undefined, faecal microbiota transplants outside the probiotic framework
6	New commensals and consortia comprising defined strains from human samples, with adequate evidence of safety and efficacy, are ‘probiotics’

Abbreviation: FAO: Food and Agriculture Organization of the United Nations.

In our invitation to the Research Topic, we invited scientists to discuss the Applications of Probiotics. When the submission was closed four papers were published on the topics, *Enterococcus faecium*, probiotics and gut-brain axis, probiotics and alcohol related liver disease and microplastics and probiotics.

Modern classification techniques of Enterococci resulted in the transfer of some members of genus *Streptococcus*, Lancefield's group D streptococci, to the new genus *Enterococcus*. The genus *Enterococcus* is considered as the third-largest genus in lactic acid bacteria and numerous probiotic species have been identified [15] as well as *E. faecium* [16]. One study in the Research Topic “Applications of Probiotics” by Hussain et al. [17] investigated the *in vitro* safety, tolerance capacity, aggregation potential, enzyme production, effect of different substances, antioxidant potential, and binding assays of *E. faecium*. By elucidating these properties will not only aid their probiotic potential but may also enhance their industrial applications as probiotic supplementation. In this study, the probiotic potential, postbiotic production, antioxidant activities, aggregation properties, and functional characterization of six *E. faecium* strains from biobank, Karachi, Pakistan was investigated. The selected strains revealed significant probiotic potential, with stress tolerance, aggregation, and postbiotic production. They were free from biogenic amines while exhibiting notable free radical scavenging and reducing activities. Additionally, their ability to adhere to fibrinogen and mucin indicates enhanced potential for mucosal colonization, competitive exclusion of pathogens, and improved host interaction. The ability of *E. faecium* strains to digest raffinose, a trisaccharide commonly reported in plant-based foods like beans, cabbage, and broccoli, is not digested by humans due to the absence of α-galactosidase enzymes and leading to digestive discomfort including gas production, represents a desirable probiotic trait, contributing to improving the digestion and reducing the GI tract discomfort. Two strains (*E. faecium* Se142 and *E. faecium* F25) produced slime, tested on MRS agar after two days incubation. The influence of digestive enzymes on enterocins, the production of arginine hydrolases, and the impact of glycine, arginine, and glucose on their growth performance reflected positive attributes. These findings indicate their potential as probiotic, with intended food and biotechnological applications. However, genomic and *in vivo* validation studies need further investigations.

The gut microbiome is a key player in the gut-brain axis and acts as a control element within the gut. It interacts with diet-derived nutrients, environmental factors, and probiotics. These interactions influence the balance between beneficial and harmful microorganisms and affect various physiological functions of the host. A diverse and balanced microbial community, often supported by probiotic consumption, is essential for maintaining gut homeostasis, healthy digestion, and stable emotional and cognitive states [8]. These effects are achieved through metabolic pathways involving metabolites, Toll-like receptor signaling, endocrine mechanisms, and neural communication routes. The study of Vijayaram et al. [18] recognizes the gut microbiome as a major regulator of digestion, immunity, neurotransmission, and brain health. It further shows that probiotics, especially psychobiotics, benefit these systems by restoring microbial diversity, strengthening gut barrier integrity, reducing inflammation, and promoting the production of GABA and serotonin. Consequently, these effects improve regulation of the HPA axis, enhance neurotrophic factors such as BDNF, and increase neuroprotection. The study also demonstrates that gut dysbiosis disrupts these pathways, leading to neuroinflammation, abnormal stress responses, and impaired gut–brain signaling, linked to conditions like Alzheimer's disease, Parkinson's disease, multiple sclerosis, autism spectrum disorder, depression, and anxiety. Both animal models and clinical data indicate that treatment with specific probiotic strains can improve cognitive function, reduce stress-related behaviors, and alleviate symptoms of neurodevelopmental and neurodegenerative disorders, though mechanisms and long-term effects vary by strain and require further investigation. These findings highlight the importance of a healthy gut microbiome for proper brain function and emotional regulation, and they emphasize the promising potential of targeted probiotic therapies as adjuncts in treating neurological and psychiatric conditions.

Alcohol-related liver disease (ARLD) refers to liver damage caused by excess alcohol intake. In their Editorial Commentary, Testino and Caputo [19] stated that ARLD cause 36% of cases of cirrhosis in the United States. Successful treatment for ARLD often depends on whether someone is willing to stop drinking alcohol and make changes to their lifestyle. When discussing alcohol intake, it is important to notice that intake can lead to modulation in the gut microbiota even before liver disease development [20]. As probiotics, prebiotics, and postbiotics may protect the liver through the gut-liver axis, and in the third study of the Research Topic, Pisarello et al. [21] focused on probiotics as promising therapeutics in alcohol related liver disease. Dysbiosis of the gut microbiota has emerged as a critical factor in the pathogenesis of alcohol-associated liver diseases, as alcohol consumption alters microbial composition and increases intestinal permeability, which contributes to systemic inflammation and liver injury through the translocation of endotoxins. In this regard it is important to notice the therapeutic potential of probiotics to restore microbial balance and enhance intestinal barrier function, by improving liver enzymes and reducing inflammation. In their study, the authors underscores the importance of a multifaceted approach toward understanding alcohol-associated liver diseases and the therapeutic potential of modulating the gut-liver axis through microbiota-targeted strategies and further pointed out that further research needs to elucidate optimal dosing strategies and long-term efficacy.

Plastics have outgrown most man-made materials and have long been under environmental scrutiny, and microplastics (MPs) has been a hot scientific topic since the first study was published in 1986 [22], and according to Web of Science approximately 30.000 papers are published using the key word microplastics. MPs are synthetic polymer particles smaller than 5 millimeters, and they are ubiquitous in terrestrial, freshwater, and marine ecosystems and increasingly in the human food chain [23]. They are persistent environmental pollutants with growing relevance to human health, and when ingested, MPs interact with the GI tract, where they may persist, translocate, or interfere with the host's biology, and can disrupt GI integrity, alter the microbiota composition, and provoke oxidative and inflammatory responses. For example, a Chinese study evaluated the effect of microplastics and disturbance of the gut microbiota in preschool children in Xiamen [24]. Probiotics, known for their gut health benefits, are being explored for their ability to mitigate these effects. In the review paper of Demarquoy [25] the author discussed evidence from *in vitro* and *in vivo* studies on how MPs impact the probiotic viability, adhesion, and biofilm formation, and how certain strains may counter MP-induced toxicity by modulating oxidative stress, immune function, and the epithelial barrier integrity. Regarding the concerns that MPs in the GI tract may hamper the colonization efficiency and persistence of probiotics, thus potentially diminishing their therapeutic efficacy, while *in vitro* experiments have demonstrated that *Lactiplantibacillus plantarum* and *Bacillus subtilis* creased biofilm formation when exposed to MPs. Furthermore, the manuscript discusses emerging applications in environmental microbiology, such as the potential use of native and engineered probiotics for microplastic bioremediation. Although current data highlight promising avenues, key gaps remain in our understanding of strain-specific mechanisms, long-term efficacy, and real-world applicability. By addressing these key gaps will be essential to advance probiotic-based strategies in human contexts and optimize probiotic interventions against MP toxicity.

## Use of AI tools declaration

The authors declare they have not used Artificial Intelligence (AI) tools in the creation of this article.
